# Editorial: Natural products from plants or microorganisms for treatment of non- communicable diseases

**DOI:** 10.3389/fchem.2024.1496038

**Published:** 2024-10-01

**Authors:** Sakina Yagi, Dominique Laurain-Mattar, Gokhan Zengin

**Affiliations:** ^1^ Department of Botany, Faculty of Science, University of Khartoum, Khartoum, Sudan; ^2^ Université de Lorraine, INRAE, LAE, Nancy, France; ^3^ Department of Biology, Science Faculty, Selcuk University, Konya, Türkiye

**Keywords:** natural products, non-communicable diseases, bioactive molecules, antiinflamation, Wnt inhibitory, bone tissue engineering

Noncommunicable diseases (NCDs) are accelerated by the negative impact of globalization and impose enormous human, social, and economic costs. Historically, strategies to prevent and treat communicable diseases have been a priority in many countries. This prioritization has reduced the burden of communicable diseases worldwide, but has resulted in noncommunicable diseases being largely neglected. The WHO estimates that NCDs account for 80% of the global disease burden and approximately 71% of all deaths are due to NCDs. Plants and microorganisms have the ability to produce a wide variety of bioactive molecules with therapeutic potential. They remain a leading source of novel bioactive molecules that are critical to drug discovery and biotech development. In this context, on 27th November 2023, we launched our Research Topic and invited researchers to engage in articles on natural products (molecules/extracts) with interesting biological activities related to non-communicable diseases. Frontiers in Chemistry published 4 contributions to the Research Topic, involving 35 authors from 9 countries. This editorial summarizes these articles in the following sections and we believe they will appeal to a broad readership in the field.

Inflammation is a biological response of the immune system to harmful stimuli, such as pathogens, damaged cells, or irritants (Aghasafari et al., 2019). It is a complex process aiming to remove the offending agent and repair any tissue damage (Liu et al., 2021). However, if inflammation persists or becomes chronic, it can lead to impaired function, and various diseases, such as arthritis, diabetes, heart disease, or cancer (Furman et al., 2019). Therefore, controlling and balancing inflammation is important for maintaining health and preventing chronic conditions. Cyclooxygenase-2 (COX-2) is a crucial enzyme that catalyzes the rate-limiting stages in the conversion of arachidonic acid to prostaglandins (Cao and Prescott, 2002). The most often prescribed drugs now for the treatment of inflammatory illnesses by targeting COX-2 are both traditional and modern NSAIDs (Everts et al., 2000). However, because of the NSAIDs’ negative cardiovascular side effects it is of utmost important to find new safer and effective anti-inflammatory agents (Desai et al., 2018). Two studies to this Research Topic report the characterization of bioactive molecules derived from plants with significant anti-inflammatory activity. The article from Haque et al. evaluated the anti-inflammatory effect of d-pinitol, a naturally occurring inositol, in a chick model and they also performed an *in silico* studies to demonstrate the molecular interactions of d-pinitol with COX-2. The article from Islam et al. demonstrated the anti-inflammatory effects of thymol, natural monoterpene phenol, on Swiss mice and chick models and its interaction with COX-2 via molecular docking and molecular dynamic simulations. The two molecules (d-pinitol and thymol) were found to significantly reduce inflammatory parameters (number of paw licks and edema diameter) in a dose-dependent manner and were more effective in combination with celecoxib or ketoprofen. *In silico* studies showed that these two molecules have significant binding capacity with COX-2 via conventional hydrogen bonds.

In this Research Topic, we also expanded our knowledge on the effectiveness of plant extracts in regenerating and healing bone tissue. In fact, conventional graft-based approaches like using metal/ceramic prostheses, allogenic, and autologous grafts represent many shortcomings and limitations like the need for multiple surgical interventions, elevated risk of immune rejection of non-autologous grafts, insufficient graft supply for extensive segmental bone loss, donor site pain/discomfort (Wickramasinghe et al., 2022). Indeed, the incorporation of plant extracts into polymeric scaffold materials stimulate osteogenesis and offers a potential solution to enhance bone healing and regeneration (Djehiche et al., 2024). An interesting study by Abdelaziz et al. investigated the potential of seeds of flax (*Linum usitatissimum*) as a promising material in bone tissue engineering. They have shown that hydroethanolic (70%) extract of flaxseed enhances the MG-63 osteoblast-like cells migration and differentiation on nanofiber scaffolds and significantly upregulating the RUNX2, COL1A1, and OCN genes. Accordingly, the authors suggested that flaxseed could be an interesting material in bone tissue engineering.

The advancement of computational annotations methods in metabolomics allowed researchers to evaluate a large number of natural products Research Topic and hence discover structurally novel molecules (Quiros-Guerrero et al., 2022). A study contributed by Quiros-Guerrero et al. to this Research Topic highlights the potential of combining Inventa, a metabolomics bioinformatic workflow designed to streamline the natural selection process, with bioactivity screening results to identify molecules capable of regulating the Wnt signaling pathway. The Wnt signaling pathway (Q155769) is critical in several biological processes like embryonic development, tissue homeostasis, and cellular proliferation and when dysregulated, it could lead to several disorders, including cancer (Liu et al., 2022). From the 1,600 screened compounds, 30 compounds were found with Wnt inhibitory IC_50_ ≤ 5 μg/mL and ethyl extract of the leaf of *Hymenocardia punctata* (Phyllanthaceae) was selected for further phytochemical analysis. The authors were able to isolate three known prenylated flavones and ten novel bicyclo[3.3.1]non-3-ene-2,9-diones, named Hymenotamayonins. Five of these compounds were found to exert significant Wnt inhibitory activity.

In summary, the published articles in this Research Topic demonstrate the identification of molecules or extracts from plants with interesting biological properties, allowing the understanding of the functions and mechanisms of these molecules in relation to related bioactivities and thus outlining their potential as candidates for drug discovery and bioengineering applications.
